# The effect of complementary music intervention on the patients’ quality of life after septoplasty and rhinoplasty

**DOI:** 10.1186/s12906-022-03761-4

**Published:** 2022-11-01

**Authors:** Angela Schell, Felix Wassmer, Lena Zaubitzer, Benedikt Kramer, Haneen Sadick, Nicole Rotter, Daniel Häussler

**Affiliations:** Department of Otorhinolaryngology Head and Neck Surgery, University Hospital Mannheim, Medical Faculty of the Ruprecht-Karls-University of Heidelberg, Mannheim, Germany

**Keywords:** Music intervention, Quality of life (QoL), Septoplasty, Rhinoplasty

## Abstract

**Purpose:**

Quality of life (QoL) assessment has emerged as an important evaluation tool for therapeutic treatments. The positive impact of complementary music interventions on QoL has been demonstrated in the literature, particularly in chronic and malignant diseases. However, its benefits during the perioperative period in head and neck patients have not been investigated thus far.

**Methods:**

Head and neck patients undergoing septoplasty and rhinoplasty were prospectively randomized and consecutively included in the trial. Passive music intervention (60 min per day) was applied to the intervention group. QoL was assessed using the Nasal Obstruction Symptom Evaluation (NOSE) questionnaire and the Functional Rhinoplasty Outcome Inventory 17 (FROI-17) questionnaire at three visits during the postoperative phase. Pain was measured using a visual analogue scale.

**Results:**

Forty-four patients were enrolled in the study. The NOSE score between the control group and the intervention group in the septoplasty arm differed significantly at visit #2 (p < 0.001) and visit #3 (p < 0.015). For the rhinoplasty study arm, significant differences in the FROI-17 score were also found at visit #2 and visit #3 (p = 0.04).

**Conclusion:**

Complementary music interventions can considerably improve patients’ QoL during the postoperative period. Furthermore, passive music interventions may be easily implemented in clinical practice as an additional cost-effective treatment with ubiquitous availability.

## Introduction

Diseases cause various symptoms that affect the body, mind, and constitution of individual patients. Since each patient requires a specific, individual assessment of their condition, the measurement of health-related Quality of Life (QoL) has become more important.

QoL assessment has emerged as an important tool for the evaluation of therapeutic interventions, especially chronic diseases. Some authors even insist that QoL should be pursued as the primary therapeutic goal in chronic diseases [1]. Of note, this concept contrasts with traditional therapy evaluation, as therapeutic success is not assessed by the therapist, but rather by the patients themselves [2]. One advantage of validated questionnaires is that they can better record subjective symptoms, such as breathing restrictions, anxiety, or dizziness, which cannot be objectively measured and are all different with regards to their individually perceived severity.

For many chronic diseases, the concept of QoL has gained increased acceptance in recent decades for measuring the effectiveness of therapies such as aromatherapy, manual therapy, acupuncture, or music therapy. Considerable interest in these complementary approaches, especially music therapy for cancer patients, has already been generated in survey studies [3]. Indeed, music therapy has been shown to relieve pain in chronic cancer patients and reduce the need for painkillers [4]. Furthermore, a positive effect of music interventions on patients’ health-related QoL was reported even with low additional effort and a good acceptance and tolerability by the patients [5–7]. Different beneficial effects on symptoms, such as anxiety, depression, pain, and QoL, can be achieved in cancer patients using music interventions [8, 9]. Other complementary treatment approaches such as art therapy, acupuncture, tuina, tai chi, qugong, or traditional Chinese medicine have also been described in the literature, showing positive results in regard of improving QoL in cancer patients [10, 11]. Perioperative music therapy has also been shown to reduce anxiety and pain in patients after visceral surgery [12]. In comparison to other complementary treatment approaches, passive music interventions may be easily implemented into clinical routine as a cost-effective add-on therapy. Furthermore, in the aera of smartphones and wireless internet access in public spaces, music has become an ubiquitous resource available for everyone.

To evaluate the effectiveness of music interventions, feasibility studies (especially for palliative-stage cancer patients) are available [13]. However, music as a complementary therapeutic option could also be useful in perioperative management [14]. The underlying physiological mechanisms that induce positive effects on patients’ symptoms and QoL are not yet completely understood [15, 16].

In the literature, increased activity in the mesolimbic system and the release of dopamine (an important neurotransmitter involved in the perception of positive emotions) are discussed [15]. Although this has already been investigated for patients suffering from chronic diseases, few studies have been published on QoL and complementary therapies for patients suffering from acute diseases or the perioperative phase of head and neck surgery. Procedures such as septoplasty or functional rhinoplasty are associated with a deterioration of obstructive symptoms during the postoperative period. Therefore, the assessment of QoL may be useful for evaluating the efficacy of complementary music interventions for individual patients [17].

Apart from decongestive treatments and pain medication, only little is known about the interventions that may improve quality of life in these patients. during the postoperative phase. In this study, we aimed to determine the effect of music interventions on the QoL of patients undergoing septoplasty and functional rhinoplasty.

## Materials and methods

This study was conducted in the Department of Otorhinolaryngology, Head and Neck Surgery at our university. Written informed consent was obtained from all individuals enrolled in the study. The study protocol was approved and reviewed by the local ethics committee board (reference number: 2020 − 557_1-AF5). The protocol was performed in accordance with the guidelines of the Declaration of Helsinki.

All adult patients that underwent septoplasty or functional rhinoplasty and who were able to give their written informed consent met the inclusion criteria. Exclusion criteria were defined as inability to fill out the questionnaires (e.g., due to lack of language capabilities or due to mental limitations) or inability to listen to music with headphones (e.g., deafness or harvest of ear cartilage on both ears). All included patients underwent septoplasty or functional rhinoplasty from February 2021 to November 2021, inclusive. After they had provided their written informed consent, the patients were randomized into either an intervention group or a control group. Simple randomization (flipping a coin) was used to assign each patient to a study group. The randomization was conducted by two independent persons. One person was responsible for inclusion of the patients, while the second person was responsible for randomization after anonymization. All patients in the intervention group were asked to listen to music for at least 60 min per day using headphones over a period of 14 days after surgery. The music content, the volume, type of music, and the time of start during the day was decided by the patients themselves. To prevent contamination of the two study groups, patients were asked to listen to music by headphones. Furthermore, only one patient per room was included into the study. All patients in both groups were asked to fill out questionnaires on day 1 after surgery, on the day of discharge (day 3), and 14 days after surgery. These three assessments are listed as visit #1, visit #2, and visit #3 in results that follow. One day after, the questionnaires were collected by one of the investigators.

The Nasal Obstruction Symptom Evaluation (NOSE) questionnaire, as well as a diary to document postoperative pain using a visual analogue scale, were completed by patients who underwent septoplasty. Furthermore, the patients documented the time and duration of the music interventions each day. Equally, the patients who underwent rhinoplasty were asked to fill out the same diary, but instead, quality of life was assessed using the Functional Rhinoplasty Outcome Inventory 17 (FROI-17). All patients with the three completed questionnaires were included in the statistical analysis. All patients were offered pain medication following the WHO pain ladder. Pain medication was adapted to each patient individually and pain level was assessed to clinical standards three times a day.

The statistical analysis was performed using Microsoft Excel (Microsoft, Redmond, WA) and JMP software (JMP 15; SAS Institute, Cary, NC). The *t*-test and analysis of variance (ANOVA) were used to compare the study groups and the three different questionnaires for the three visits. P-values less than 0.05 were considered significant.

## Results

**Fig. 1:**
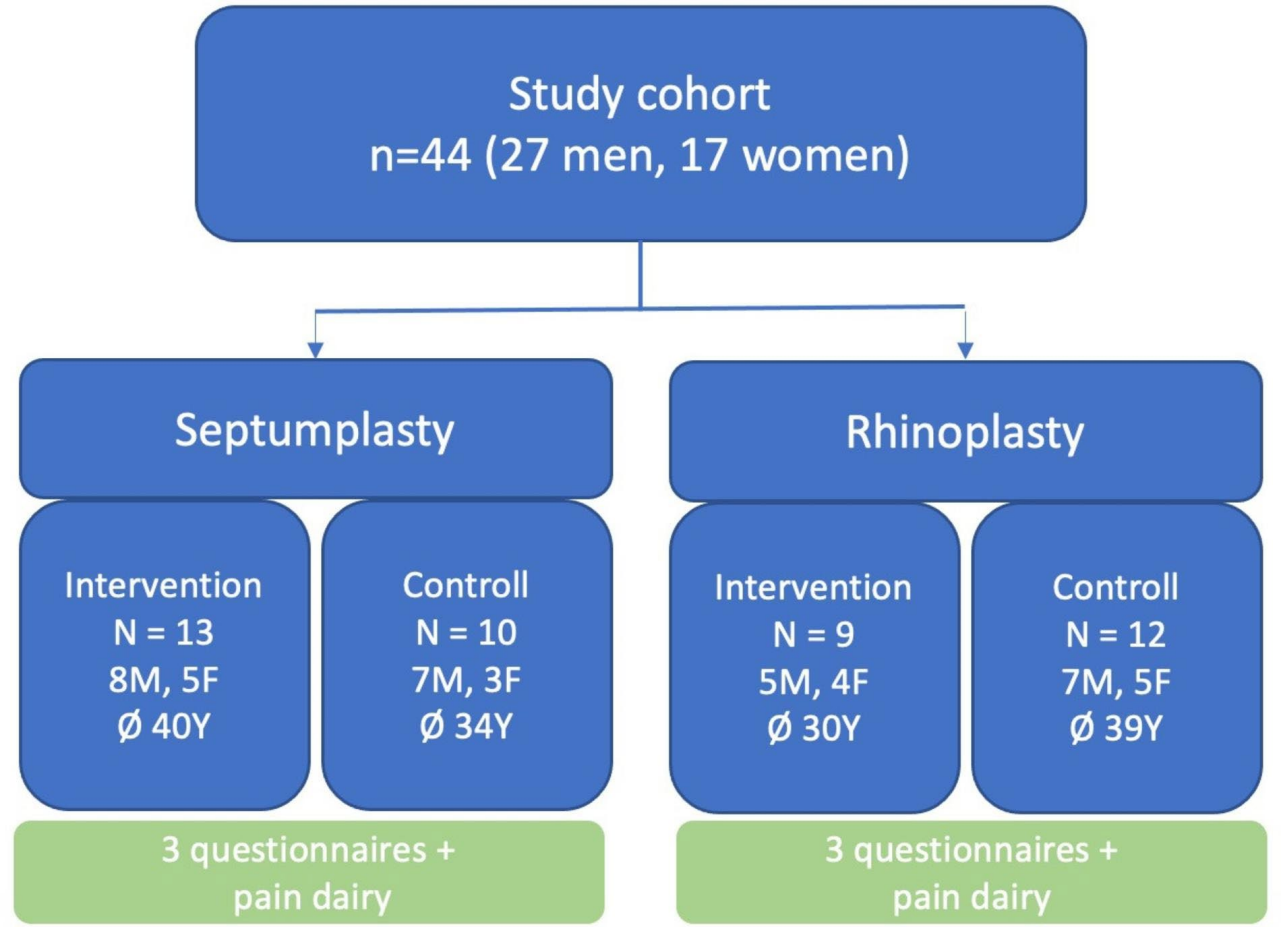
Depiction of the study cohort and both study arms

A total of 44 patients were consecutively enrolled in the study. The details of the study cohort are shown in Fig. 1. The participants consisted of 27 men and 17 women with a mean age of 36 years (range 18–76 years). All of the 44 included patients completed the questionnaires, and no patient was lost to follow-up.

### Septoplasty

A total of 24 patients (13 patients in the intervention group and 10 patients in the control group) were included in this septoplasty subgroup. The mean age of the patients was 40 years (range 18–76 years) in the intervention group and 33 years (range 20–55 years) in the control group. The patients in the intervention group listened to music for 59 min ± 7.6 min per day, on average. The mean NOSE scores in the control group were 16.8 ± 2.8 at visit #1, 13.1 ± 2.2 at visit #2, and 5.1 ± 4.0 at visit #3. The scores differed significantly between these three visits (p < 0.001).

**Fig. 2:**
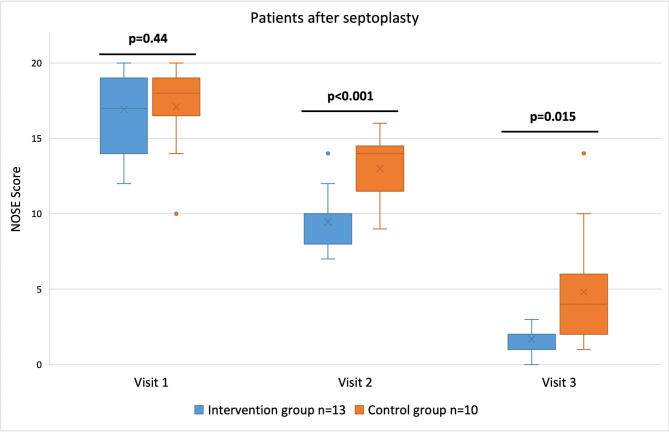
NOSE scores of patients that underwent septoplasty are shown in blue for the intervention group and in orange for the control group at the time of each visit

In the intervention group, the mean NOSE scores were 16.9 ± 2.7 at visit #1, 9.5 ± 2.0 at visit #2, and 1.7 ± 0.8 at visit #3 (p < 0.001). Comparing both groups at each visit, significant differences were found at visit #2 (p < 0.001) and visit #3 (p < 0.015); see Fig. 2 for details. A relative reduction of nasal obstruction symptoms (as measured by the NOSE score) of 27.2% and 64.9% was found by comparing both study groups at visits #2 and #3, respectively.

With regard to the content of their pain diaries, the intervention group patients documented 5.3 ± 2.4 days without any pain, and the control group patients documented 3.8 ± 2.9 days without any pain (p = 0.11); no statistically significant differences between the two groups were found. The mean pain level documented by the intervention group in their pain diaries was lower than that in the control group during the entire postoperative follow-up period.

**Fig. 3:**
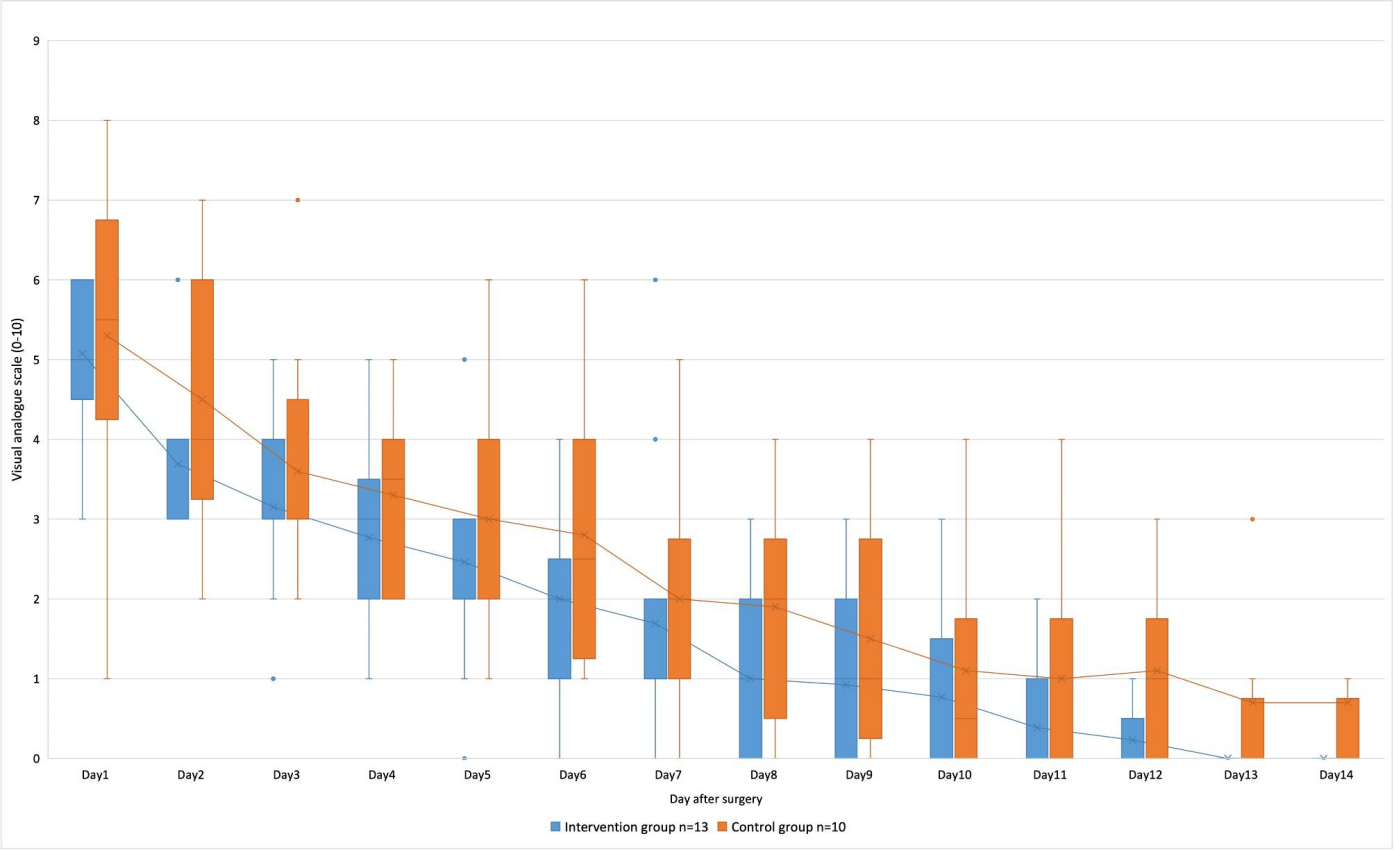
Depiction of the pain diary of patients who underwent septoplasty shown by blue for the intervention group and in orange for the control group at the time of each visit. Averages are shown by the solid line

The patients seemed to especially benefit from the music intervention during the first week, thus resulting in lower pain levels. However, no significant difference in the visual analogue scale was found when both groups were compared on each visit day (see Fig. 3).

### Rhinoplasty

In total, 21 patients were included in this arm of the study: 9 patients were included in the intervention group and 12 patients were randomized into the control group. The mean ages were 30 years (range 22–35 years) and 39 years (range 22–67 years) in the intervention group and the control group, respectively.

The mean documented duration of music intervention was 60 min ± 12 min. Mean FROI-17 scores in the intervention group were 37 ± 8 at visit #1, 24 ± 6.2 at visit #2, and 7.4 ± 8.0 at visit #3. Meanwhile, the control group recorded scores of 39.8 ± 12.1 at visit #1, 30.8 ± 10 at visit #2, and 14.8 ± 9.4 at visit #3.

**Fig. 4:**
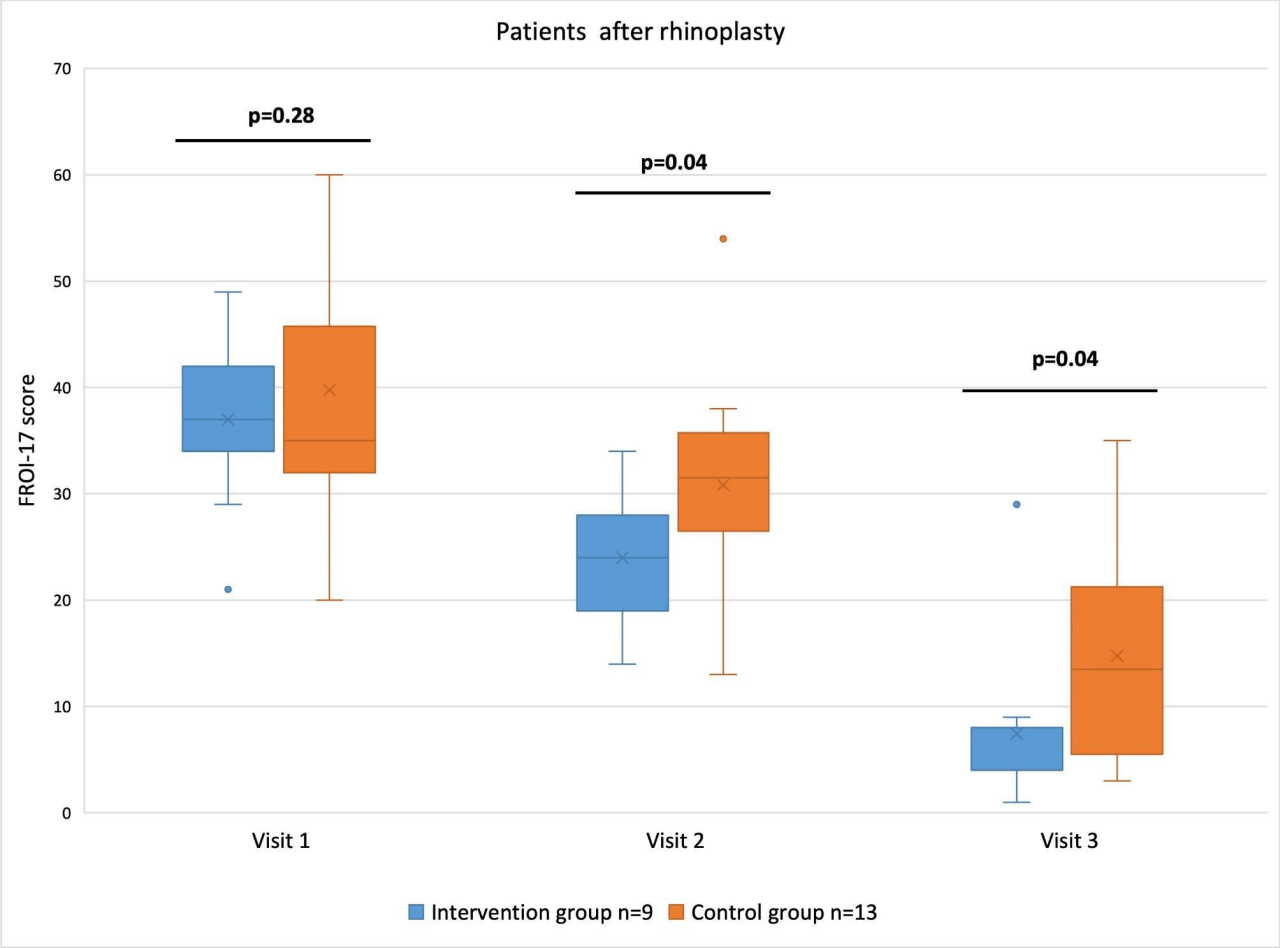
FROI-17 scores of patients who underwent rhinoplasty are shown in blue for the intervention group and in orange for the control group at the time of each visit

In both groups, the scores differed significantly between the three assessments (p < 0.001). Furthermore, as shown in Fig. 4, significantly different scores were found at visit #2 and visit #3 (p = 0.04). The relative reduction of impairment of QoL, as measured by the FROI-17 questionnaire, was 22.1% and 49.5% at visit #2 and visit #3, respectively, when the intervention group was compared to the control group.

**Fig. 5:**
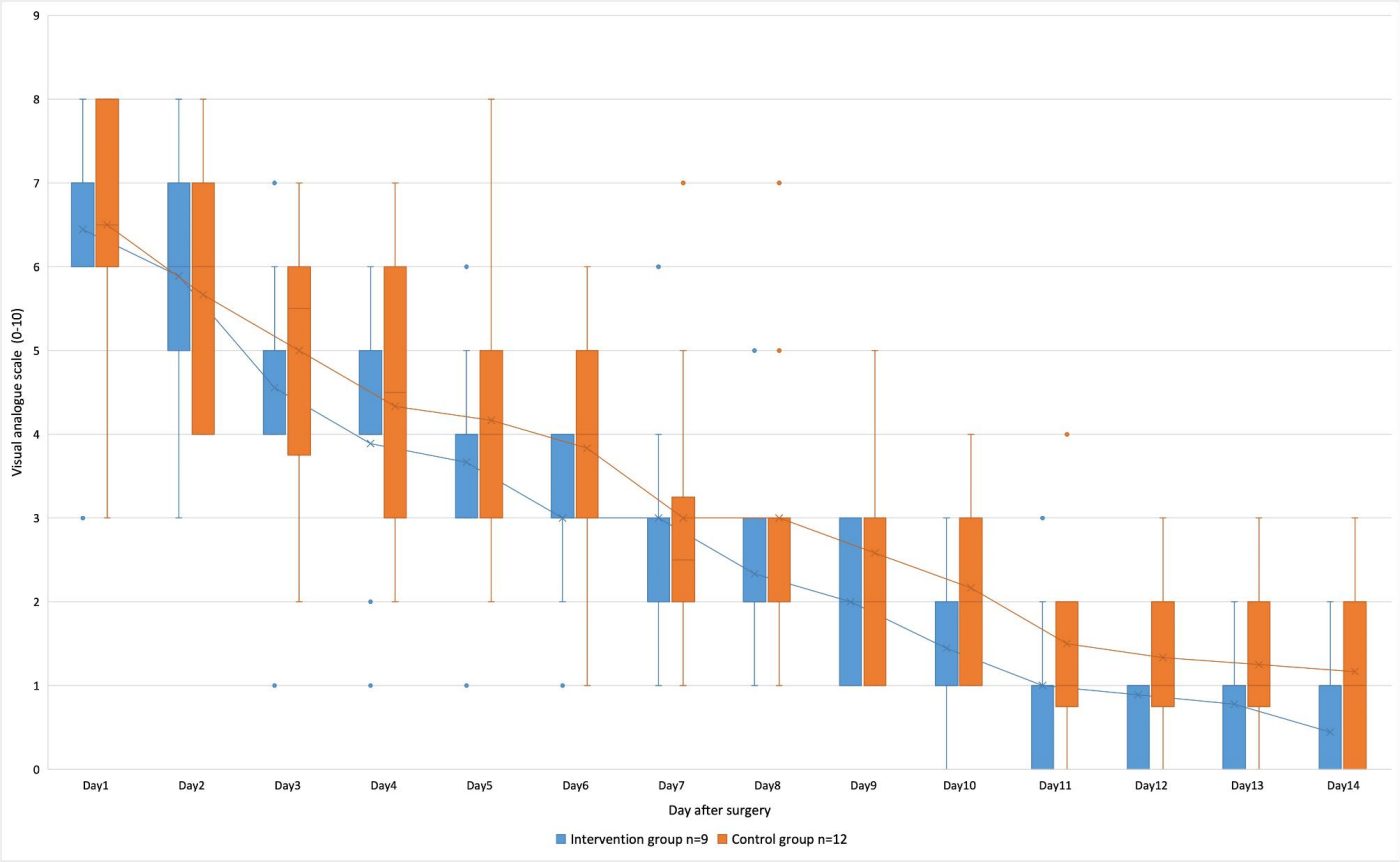
Depiction of the pain diary of patients who underwent rhinoplasty in blue for the intervention group and in orange for the control group at the time of each visit. Averages are shown by the solid line

The intervention group patients had 2.1 days ± 1.6 days without pain during the postoperative follow-up, whereas the control group patients documented 0.9 days ± 1.1 days. This result appeared to show an association; however, the result was not significant (p = 0.050). As seen in their diaries, the patients in the intervention group seemed to benefit from music intervention, especially during the first 6 days after surgery; however, the apparent association, derived from the diagram in Fig. 5, was not statistically significant.

## Discussion

Music may influence individuals in a multidimensional way as it may cause psychological and physical effects. Even though much literature has been published on the effects of music on emotional responses, including the effects on body stress levels and other measurable effects on body functions, the use of music intervention as an additional complementary treatment approach (especially in head and neck surgery) has not been investigated to date [18–21].

Suda et al. hypothesized that music may reduce stress as it induces emotional responses in the upper temporal cortex areas of the brain in a similar way to pleasant experiences or happiness [22]. Leardi et al. even found a measurable modulation of neurohormonal serum plasma levels by perioperative music intervention [23].

The beneficial effects of music interventions have been demonstrated in the literature, especially for patients suffering from cancer with regards to pain, mood, anxiety, and QoL [24–28]. Two meta-analyses found additional positive effects for the aspects of perioperative pain and anxiety [14, 28]. Patients’ interest in complementary music interventions has also been shown in a study by Gencer et al. [3], while several authors have stated that a positive impact on patients’ health-related QoL may even be achieved with low additional effort. Therefore, it seems feasible to integrate music therapy into different therapeutic regimens [5–7]. Still, music interventions, as a complementary treatment option, have not been implemented into therapy algorithms thus far.

This study aimed to investigate the effect of music intervention on QoL in patients undergoing septoplasty or rhinoplasty in a prospective randomized trial. The selected questionnaires in our study have been well evaluated and validated and are available in multiple languages [29–31]. On the basis of the available literature, Poulsen et al. recommended listening to music at least 15–30 min per session, with calming rhythms at 60 to 80 beats per minute, providing a list of available titles, and using music throughout the perioperative process [32]. As we aimed to assess the impact of music intervention on QoL, music intervention was recommended to our patients only during the postoperative phase and not intraoperatively. As pain may additionally impair patients’ QoL, this aspect was assessed separately.

In our study, we found more pain-free days in the intervention groups in both study arms, but there were no statistically significant differences. Pain was evaluated using a visual analogue scale. Although this is a common way of assessing pain in clinical studies, it may be influenced by multiple individual variables.

Different confounding factors affect pain. Patients were asked to document pain once each 24-hour day. Therefore, the actual mood or pain at this time may influence the perceived pain level at the last 24 h. Due to the relatively small sample size and the fact that patients undergoing one or both surgical procedures do not demand excessive analgesia during the postoperative phase (increasing the chances of reaching “floor effect”), the reduction of pain medication may only be assessed with statistical rigor in large sample sizes. Still, the reduction of days without any pain may indicate a reduction in pain levels caused by passive music interventions.

In both study arms, a significant impact on QoL was seen in the music intervention groups. As patients were randomly assigned to each study group, we assumed that this effect was not due to the patients’ habits of listening to music for the relief of anxiety or pain. In contrast to the recommendations of Poulsen et al., we left the choice of music to the patients themselves. In our opinion, the therapeutic effect may be compromised by offering music to patients that conflicts with their preferences.

In our study, patient adherence was good, as the recommended intervention could be achieved with very little additional effort. As complementary music interventions are unlikely to have any adverse side effects, and because smartphones and music streaming are freely available and have become ubiquitous, it seems plausible to transfer our findings to clinical practice.

To our knowledge, this is the first prospective randomized study trial showing improvement in QoL via a passive music intervention in patients undergoing septoplasty or rhinoplasty. To evaluate whether music interventions improve postoperative pain or reduce the need for analgesic medication in rhinoplasty and septoplasty, larger cohorts in future trials are needed.

## Data Availability

The datasets generated and analysed during the current study are not publicly available, as they are only stored on a local drive due to privacy regulations but are available from the corresponding author on reasonable request.
